# Prediction of Lower Urinary Tract, Sexual, and Bowel Function, and Autonomic Dysreflexia after Spinal Cord Injury

**DOI:** 10.3390/biomedicines11061644

**Published:** 2023-06-06

**Authors:** Chiara Pavese, Thomas M. Kessler

**Affiliations:** 1 Department of Clinical-Surgical, Diagnostic and Pediatric Sciences, University of Pavia, 27100 Pavia, Italy; chiara.pavese@unipv.it; 2 Istituti Clinici Scientifici Maugeri IRCCS, Neurorehabilitation and Spinal Unit of Pavia Institute, 27100 Pavia, Italy; 3 Department of Neuro-Urology, Balgrist University Hospital, University of Zürich, 8008 Zürich, Switzerland

**Keywords:** rehabilitation, spinal cord injuries, prognosis, neurogenic lower urinary tract dysfunction, neurogenic sexual dysfunction, neurogenic bowel, autonomic dysreflexia, outcome assessment

## Abstract

Spinal cord injury (SCI) produces damage to the somatic and autonomic pathways that regulate lower urinary tract, sexual, and bowel function, and increases the risk of autonomic dysreflexia. The recovery of these functions has a high impact on health, functioning, and quality of life and is set as the utmost priority by patients. The application of reliable models to predict lower urinary tract, sexual, and bowel function, and autonomic dysreflexia is important for guiding counseling, rehabilitation, and social reintegration. Moreover, a reliable prediction is essential for designing future clinical trials to optimize patients’ allocation to different treatment groups. To date, reliable and simple algorithms are available to predict lower urinary tract and bowel outcomes after traumatic and ischemic SCI. Previous studies identified a few risk factors to develop autonomic dysreflexia, albeit a model for prediction still lacks. On the other hand, there is an urgent need for a model to predict the evolution of sexual function. The aim of this review is to examine the available knowledge and models for the prediction of lower urinary tract, sexual, and bowel function, and autonomic dysreflexia after SCI, and critically discuss the research priorities in these fields.

## 1. Introduction

Persons affected by a spinal cord injury (SCI) experience a wide range of impairment of body structures and functions derived from the damage to motor, sensory, and autonomic pathways. As a consequence, a reduction in independence in daily life activity, a limitation of vocational and social participation, and a deterioration of quality of life are observed. After SCI onset, patients face up to a few months of inpatient rehabilitation, during which a multidisciplinary team takes charge of various aspects of clinical, functional, and psychological needs and works in synergy with patients and caregivers for the recovery of the maximum level of independence possible for each patient [1]. The treatment of lower urinary tract, sexual, and bowel dysfunction and the prevention of related complications, such as autonomic dysreflexia, represent a milestone in the rehabilitative care of patients with SCI, since these aspects strongly impact an individual’s health and quality of life. 

In this context, the prediction of lower urinary tract, sexual, and bowel function, and autonomic dysreflexia represents a key aspect of the correct development of the rehabilitation phase and has strong implications for the planning of future clinical trials aiming to evaluate the effect of new interventions for patients with SCI. The aim of this review is to examine the available knowledge and models for the prediction of lower urinary tract, sexual, and bowel function, and autonomic dysreflexia after SCI and critically discuss the research priorities in these fields.

### 1.1. Spinal Cord Injury

The term SCI refers to damage to the spinal cord as a consequence of trauma or disease. Epidemiological data are subjected to high variability in different geographic areas. Traumatic SCI affects 16–54 new individuals per million each year in Western countries, with a four times higher incidence in males and an observed increase in age at injury over the last decades [2,3,4,5,6]. Concerning non-traumatic SCI, there is a paucity of high-quality studies: the estimated incidence ranges from 6 to 76 cases per million individuals/year [7]. The commonest cause of non-traumatic SCI in developed countries is represented by degenerative myelopathy [7,8]. Other etiologies of SCI include ischemia, other vascular causes, tumors, infections, inflammatory/autoimmune diseases, and neural tube disorders [7]. It is expected that in the next decades, the epidemiological relevance of non-traumatic SCI will increase, due to population aging and the amelioration of the level of care in different medical and surgical areas, which implies a higher rate of patients surviving with a non-traumatic SCI [7].

The spinal cord damage causes an alteration of motor, sensory, and autonomic pathways with consequent impairment of functions below the level of injury. The clinical presentation varies depending on the level and grade of injury [2]. Impairment or loss of sensory-motor function below the level of injury reduces the patient’s motility, implying a loss or impairment of upper limb function (in patients with lesions at the first thoracic neuromere or above) and ambulation [2]. In addition, the damage of somatic and autonomic pathways may determine an alteration of cardiorespiratory function, secondary immunodeficiency, pressure sores, neurogenic heterotopic ossification, neuropathic pain, dysphagia, dysphonia, and dangerous impairments of genitourinary and gastroenteric function, i.e., neurogenic lower urinary tract, sexual, and bowel dysfunction, and autonomic dysreflexia [2].

### 1.2. Classification of Spinal Cord Injury

The neurological assessment and classification are based on the International Standards for Neurological Classification of Spinal Cord Injury (ISNCSCI), a consolidated evaluation system proposed by the American Spinal Injury Association and validated for patients with traumatic and non-traumatic SCI [9,10,11]. The ISNCSCI integrate the assessment of five key muscle strengths for each limb, the evaluation of light touch (LT) and pin-prick (PP) sensation in each body dermatome, and the presence of voluntary anal contraction and deep anal pressure. The ISNCSCI provides a few cumulative scores. Muscle function is rated on a six-level scale of strength from 0 (total paralysis) to 5 (normal strength). The upper extremity motor score (UEMS) derives from the sum of the upper limbs’ key muscles’ strength (elbow flexors, wrist extensors, elbow extensors, finger flexors, finger abductors) and ranges from 0 to 50. The lower extremity motor score (LEMS) derives from the sum of the lower limbs’ key muscles’ strength (hip flexors, knee extensors, ankle dorsiflexors, long toe (big toe) extensors, ankle plantar flexors) and ranges from 0 to 50. The total motor score derives from the sum of UEMS and LEMS and ranges from 0 to 100. Sensory function is evaluated through LT and PP sensation in each dermatome and rated as absent (grade 0), altered (grade 1), or normal (grade 2). LT and PP scores result from the sum of all dermatome grades ranging from 0 to 112. The ISNCSCI allows the definition of the neurological level of injury (NLI) and of the American Spinal Injury Association Impairment Scale (AIS) grade. The NLI refers to the most caudal spinal cord segment with intact sensation and antigravity muscle function strength in the presence of intact rostral sensory and motor function. The AIS grade defines the degree of completeness and reflects the severity of SCI: it ranges from A (complete impairment) to E (normal function).

To investigate the impact of autonomic nervous system impairment on body structures and functions, the International Standards to document Autonomic Function following SCI (ISAFSCI) is recommended [12]. This tool displays a section specifically dedicated to autonomic and somatic control of sacral function and includes both neurologic and subjective assessments related to the lower urinary tract, sexual, and bowel function.

The evaluation of independence in daily life activities may be performed through the Functional Independence Measure (FIM) [13,14], or through the Spinal Cord Independence Measure (SCIM) [15]. FIM is a non-disease-specific tool, which investigates independence in daily life activities including self-care, sphincter control, transfers, ambulation, communication, and social cognition. Items G and H are dedicated to the evaluation of independence in the lower urinary tract and bowel outcomes, respectively. Each item may be scored on a seven-level scale, based on the amount of assistance required to perform each activity (from 1 = total assistance required to 7 = total independence). SCIM is a tool specifically developed to evaluate independence in daily life activities for patients with SCI. The domains investigated include self-care, respiration, sphincter management, and mobility. Items 6 and 7 of SCIM are dedicated to the evaluation of lower urinary tract and bowel management, respectively. Item 6 is rated on a seven-level scale, with a score ranging from 0 (indwelling catheter) to 15 (post-void residual <100 mL; continent; does not use external drainage instrument) [15]. Item 7 is rated on a four-level scale, with a score ranging from 0 (irregular timing or very low frequency (less than once in three days) of bowel movements) to 10 (regular bowel movements, without assistance; no accidents) [15]. However, neither FIM nor SCIM investigates sexual function.

## 2. Genitourinary and Gastroenteric Impairments and Priority of Recovery

### 2.1. Neurogenic Lower Urinary Tract Dysfunction

The function of the lower urinary tract depends on the coordinated activity of smooth and striated muscles, which ensure the regular alternation of storage (urinary bladder) and emptying (bladder neck, urethra, and urethral sphincter) phases [16]. The coordination of this function is mediated by a complex neural control distributed across central pathways (brain and spinal cord) and peripheral pathways (pelvic parasympathetic, thoracolumbar sympathetic, and somatic fibers) [12,16]. A lesion located at the infrapontine-suprasacral level produces an alteration of lower urinary tract function that involves both storage and voiding phases [17]. Urodynamics is the gold standard instrumental evaluation to assess detrusor and bladder outlet function as well as detrusor pressure and compliance [17]. The great majority of patients with SCI develop a neurogenic lower urinary tract dysfunction and 90% of patients show at least one unfavorable urodynamic parameter within the first year after traumatic or ischemic SCI [18]. Immediately after SCI, loss of voluntary and supraspinal control may lead to an acontractile/hypocontractile detrusor [19]. After the phase of spinal shock, a progressive recovery of spinal reflexes is usually observed, with the development of automatic micturition, a typical pattern of detrusor overactivity, and detrusor–sphincter dyssynergia [16]. As a consequence of this disequilibrium, the patient may experience urinary urgency, increased frequency, nocturia, incomplete voiding, and incontinence. If not adequately diagnosed and treated, this condition may lead to increased detrusor pressures, with the consequent risk of morphological changes in the bladder wall, vesico-ureteral-renal reflux, urinary tract infections, hydronephrosis, renal impairment, autonomic dysreflexia, and eventually end-stage renal disease [17].

### 2.2. Neurogenic Sexual Dysfunction

Sexual function is frequently and dramatically affected after SCI. This impairment derives from a complex interplay among bio-psycho-social factors [20]: direct damage of spinal pathways contributing to the regulation of sexual function (pelvic parasympathetic, thoraco-lumbar sympathetic, and somatic motor descending and sensory ascending components), SCI complications (spasticity, pressure sores, urinary or fecal incontinence, autonomic dysreflexia), psychological and cultural factors, and medication side effects [21]. Patients with SCI experience sexual dysfunction involving different domains: desire, arousal, orgasm, ejaculation, and fertility [20]. It is reported that 50% of women and 29% of men report impaired sexual desire, 70–81% of men have erectile dysfunction, 50–80% of women experience reduced arousal or vaginal dryness, almost all male patients report impaired or absent ejaculation, and 75–89% have orgasmic dysfunction [20]. Impairments of sexual functions may reduce fertility in male patients as a consequence of alteration of ejaculation and semen [22]. Pregnancy is still possible for female patients with SCI, but they may face specific complications during pregnancy, including autonomic dysreflexia [22]. It is important to highlight that there is growing evidence of an inadequate level of sexual counseling and rehabilitative programs for patients with SCI, despite the crucial importance of sexual function for the patient’s and partner’s health and quality of life [23,24].

### 2.3. Neurogenic Bowel Dysfunction

The loss of control over autonomic and somatic pathways is responsible for the onset of neurogenic bowel dysfunction after SCI. The clinical presentation includes alterations in colon motility and impairment of anorectal sphincter function, resulting in bowel imbalance, alterations in mucosal secretions, and vascular tone [25,26]. Patients may experience constipation, fecal incontinence, or a combination of the two symptoms. This condition has several complications, which may expose patients to potentially life-threatening sequelae, including abdominal pain, rectal bleeding, rectal prolapse, anal fissure, bloating, nausea, autonomic dysreflexia, prolonged evacuation, impaction, and intestinal obstruction up to the mechanical ileus [26]. To date, there is limited evidence concerning the therapeutic approaches for neurogenic bowel dysfunction, and in most cases, different interventions are used in sequence or combination based on empirical approaches [27]. The lack of high-quality studies in this field may partially explain the diffuse failure of therapeutic interventions for bowel function. Overall, 42% of patients report severe bowel symptoms, and subjects with complete lesion display slower colonic transit time resulting in more severe clinical presentation compared to patients with incomplete injuries [25,28]. It is reported that 22% and 14% of adult patients with acquired SCI require more than 30 and 60 min to complete evacuation, respectively [29]. Moreover, 12% of patients require help for evacuation and 23% are completely dependent [29].

### 2.4. Autonomic Dysreflexia

Autonomic dysreflexia is a potentially life-threatening complication of SCI which mainly affects patients with a NLI at T6 or above [30]. This syndrome derives from a dysregulation of the autonomic system and appears as a sudden increase of systolic blood pressure (≥20 mmHg) in consequence of an aspecific trigger below the level of injury [31]. In most cases, the trigger stimulus originates from the genitourinary or gastrointestinal tract, e.g., urinary retention or infection, sexual activity, constipation, hemorrhoids, or fissure [30,32]. Moreover, cystoscopy and urodynamic investigation may trigger this syndrome [33]. Through afferent sensory fibers, the aspecific stimulus triggers activation of sympathetic neurons located between T5 and L2, leading to hypertension and pallor, cool skin, and piloerection below the level of the lesion [30]. In response to a blood pressure increase, carotid and aortic baroreceptors activate a parasympathetic response, leading to bradycardia and brainstem-mediated peripheral vasodilation, resulting in headache, blurred vision, nasal congestion, and flushing with sweating above the level of injury [30]. These compensatory responses, however, are not able to reach the districts below the level of injury due to the disconnection of spinal sympathetic centers from supraspinal control as a consequence of SCI. Therefore, the uncontested sympathetic activity persists below the level of injury, thus maintaining hypertension [32]. This condition requires a prompt treatment consisting of the removal of trigger factors and pharmacological treatment of hypertension in case of persistence of elevated values [30]. If not promptly and adequately treated, autonomic dysreflexia may lead to severe or fatal consequences, such as cerebrovascular hemorrhage or ischemia, seizures, arrhythmia, myocardial ischemia, pulmonary edema, and cardiac arrest [34].

### 2.5. Patients’ Priority for Functional Recovery

Genitourinary and gastroenteric complications of SCI have a devastating impact on patients’ health, functioning and quality of life. Complications related to neurogenic lower urinary tract dysfunction have been the first cause of death in the past [35]. Although mortality as a consequence of these complications has been progressively reduced due to the amelioration of diagnostic and management methods, these sequelae continue to be a relevant cause of morbidity [17]. Indeed, lower urinary tract infections represent common sequelae for patients with SCI, who are exposed to increased risk of sepsis and renal failure. A poor control of bowel function exposes patients to risk of constipation, malnutrition and pressure ulcers. Lower urinary tract and bowel care require in many cases a long time and a caregiver’s support [26,36]. Taking into consideration the definition of individuals’ functioning provided by the International Classification of Functioning, Disability and Health (ICF), urinary and fecal incontinence have a dramatic impact on all levels of an individual’s functioning, including damage of body function and structures, limitation in independence in daily life activity, and restriction in working capacity and social participation [25,26,37]. All these aspects contribute to a deterioration of quality of life, which is defined by the World Health Organization as “individuals’ perception of their position in life in the context of the culture and value systems in which they live and in relation to their goals, expectations, standards and concerns” [25,26,38]. The burden related to the management of genitourinary and gastroenteric dysfunction explains why the recovery of lower urinary tract, sexual, and bowel function is set as the utmost priority by patients with SCI, being for example more important than recovery of ambulation or control of pain [39,40].

## 3. Prediction of Genitourinary and Gastroenteric Outcomes

### 3.1. Prediction of Lower Urinary Tract Function

**Figure 1:**
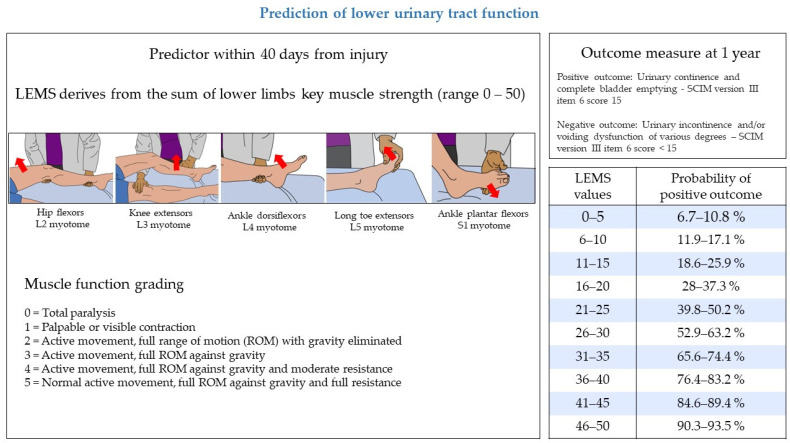
Prediction model of lower urinary tract function and expected probability of positive outcome one year after traumatic SCI. The model is based on a single predictor, the Lower Extremity Motor Score (LEMS) of the International Standards for Neurological Classification of Spinal Cord Injury (ISNCSCI). One-year lower urinary tract outcome is evaluated through a dichotomization of the Spinal Cord Independence Measure (SCIM) item 6 score. The table reports the expected probability of positive outcome at 1 year based on LEMS values collected within 40 days from injury. Images refer to the muscle strength evaluation of grade 3. Images modified from the Motor Exam Guide, American Spinal Injury Association: International Standards for Neurological Classification of Spinal Cord Injury, revised 2019, Richmond, VA with permission of the American Spinal Injury Association.

A study based on a large and prospectively-collected dataset derived from the European Multicenter Study about Spinal Cord Injury (EMSCI) identified two models to predict the recovery of lower urinary tract function one year after traumatic SCI [41]. In this study, a positive outcome at one year was defined as urinary continence (assessed by bladder diary) and complete bladder emptying (i.e., post-void residual <100 mL assessed by ultrasound or “in-out” catheterization). Lower urinary tract function and management at one year were evaluated through SCIM version II or III item 6 (sphincter management—bladder), where a score of 15 indicates a positive outcome [15,42]. The two models relied on predictors recorded within 40 days from traumatic SCI and calculated the probability of positive lower urinary tract outcome at one year, expressed in percentage. The full model relied on three clinical predictors: the LEMS of ISNCSCI, the highest score between the right and left side of the light-touch sensation in the S3 dermatome of ISNCSCI and SCIM subscale respiration, and a sphincter management score (investigating the independence in breathing, lower urinary tract, bowel, and toilet management). The simplified model relies on a single predictor, the LEMS of ISNCSCI. Figure 1 illustrates the predictor, the outcome measure, and the expected probabilities of recovery of lower urinary tract function calculated with the simplified model. The algorithm of both models is included in the original publication [41]. Both full and simplified models confirmed an excellent performance in an external validation [41]. Moreover, the validity of the simplified model based on LEMS only was confirmed by an independent study on a large dataset of patients included in the United States National Spinal Cord Injury Database [43]. The two prediction models of lower urinary tract outcome demonstrated a high performance also in a cohort of EMSCI patients with ischemic SCI, thus extending the validity of these tools also to patients with ischemic lesions [44].

A study based on data from the United States National Spinal Cord Injury Model Systems (SCIMS) Database applied artificial neural networks and logistic regression analysis to develop prediction models of functional outcomes one year after traumatic SCI, including a model to predict lower urinary tract management [45]. A positive lower urinary tract outcome was defined as modified or completely independent management, as measured by a score of 6 or 7 through the Functional Independence Measure (FIM) dedicated item [13,14]. As predictors, they used a categorization of ISNCSCI UEMS, LEMS, and binary values of C5, C6, C7, C8, L2, L3, and S1 key muscle strengths [45]. For this model, the authors suggested further refinement and validation before applying it in clinical practice [45].

Previous studies identified a few urodynamic parameters with relevance in the prediction of upper urinary tract damage, such as reduced compliance and high detrusor leak point pressure [46,47]. In most cases, unfavorable parameters are already detectable at the urodynamic evaluation in the first trimester after traumatic or ischemic SCI [18,48,49]. Future integration of urodynamic data in clinical models may optimize their predictive capacity.

### 3.2. Prediction of Sexual Function

Sexual and reproductive activities are frequently damaged as a consequence of SCI, and the identification of preserved functions plays a key role in the definition of residual resources that serve as a basis for rehabilitation [21]. In this context, a careful assessment of somatic and autonomic pathways is mandatory [50] and is relevant in predicting sexual function recovery [50]. In addition, the evaluation of comorbidities and medications that may have an impact on sexual function is necessary [20]. The NLI and degree of lesion completeness are considered important predictors of sexual function recovery [21]. After complete SCI above T10, preservation of reflexogenic arousal is expected but the interruption of the connection between the brain and the thoracic-lumbar spinal center implies a loss of psychogenic arousal [21]. On the other hand, in the presence of complete damage at the sacral level, the reflexogenic pathways are damaged, but the psychogenic arousal is maintained [21]. Preservation of combined light touch and pinprick sensation within the T11-L2 dermatomes predicts the possibility of psychogenic arousal, while the presence of bulbocavernosus reflex is predictive of reflexogenic genital arousal [21,51]. Despite the enormous impact of sexual function on quality of life, to date, there is no model to predict the recovery of sexual activity, probably due to the complex interplay among bio-psycho-social factors implicated in this function [20,21]. Further studies are needed to optimize both pharmacological and non-pharmacological interventions and to promote the development of reliable prediction models of sexual function to be employed both in clinical and research contexts [52].

### 3.3. Prediction of Bowel Function

**Figure 2:**
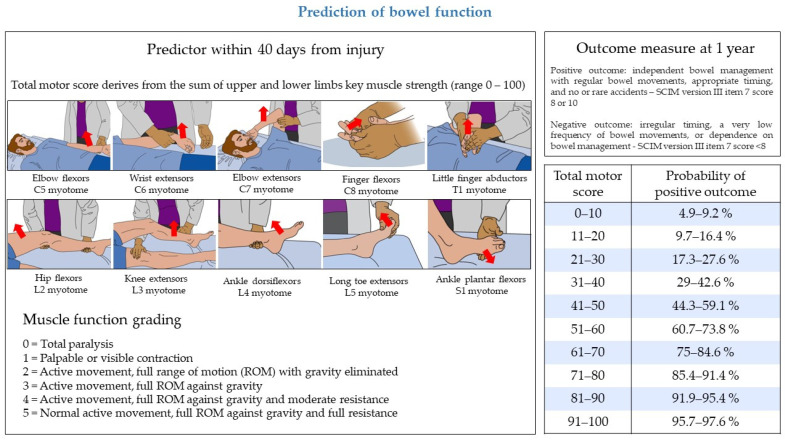
Prediction model of bowel function and expected probability of positive outcome one year after traumatic SCI. The model is based on a single predictor, the total motor score of the International Standards for Neurological Classification of Spinal Cord Injury (ISNCSCI). One-year bowel outcome is evaluated through a dichotomization of the Spinal Cord Independence Measure (SCIM) item 7 score. The table reports the expected probability of a positive outcome at 1 year based on the total motor score collected within 40 days from injury. Images refer to the muscle strength evaluation of grade 3. Images modified from the Motor Exam Guide, American Spinal Injury Association: International Standards for Neurological Classification of Spinal Cord Injury, revised 2019, Richmond, VA, with permission of the American Spinal Injury Association.

A model to predict bowel function one year after traumatic SCI was derived from a large and prospectively-collected EMSCI dataset [53]. The model defined a positive bowel outcome as independent management with regular bowel movements and appropriate timing, with no or rare accidents (i.e., fecal incontinence less than twice a month). A positive outcome corresponded to a item 7 (sphincter management—bowel) score of 10 points in SCIM version II and of 8 or 10 points in SCIM version III [15,42]. The model relied on a single predictor, the total motor score of ISNCSCI (range 0–100). The algorithm of the model is included in the original publication [53]. Figure 2 illustrates the predictor, the outcome measure, and the expected probabilities of recovery of bowel function. The validity of the model was confirmed by an independent prospective EMSCI validation [53] and by two external validations [54,55]. A subsequent study reaffirmed the prognostic value of total motor score for the recovery of bowel function, not only when used as an early predictor, but also when evaluated three months after traumatic SCI, a time-point coinciding in many cases with the end of the rehabilitation phase [56]. Moreover, the original model showed a good performance in the prognostication of bowel function also in patients with SCI of ischemic etiology [57].

In the previously mentioned study based on data from the United States National Spinal Cord Injury Model Systems (SCIMS) Database, the authors also derived a model to predict bowel management one year after traumatic SCI [45]. The model prognosticated the probability to reach a modified or completely independent bowel management, corresponding to a value of 6 or 7 for the FIM-dedicated item, based on a categorization of LEMS and on a binary value of strength at C5, C6, C7, C8, and T1 myotomes [45]. Also for this model, further refinements and validations were suggested by the Authors [45].

### 3.4. Prediction of Autonomic Dysreflexia

Despite the threat represented by this condition, autonomic dysreflexia remains underdiagnosed [33,58]. Accurate data on predictors of autonomic dysreflexia derive from studies investigating the onset of autonomic dysreflexia during the urodynamic investigation, highlighting an incidence of this complication in about two-thirds of patients with neurogenic lower urinary tract dysfunction after SCI [33,59]. Urodynamics is the gold standard examination to assess neurogenic lower urinary tract dysfunction in patients with SCI and the execution of this essential examination represents in itself a possible trigger stimulus of autonomic dysreflexia, secondary to bladder filling [32,33]. Therefore, for the execution of this procedure, continuous cardiovascular monitoring is prescribed [33]. Studies conducted during urodynamics investigation under continuous cardiovascular monitoring allowed a sensitive identification also of subclinical episodes of autonomic dysreflexia [33,59]. These studies identified two predictors of autonomic dysreflexia: level of injury at T6 or above and the presence of neurogenic detrusor overactivity [33,59]. Considering the NLI, the risk of autonomic dysreflexia is six to seven times higher in patients with a lesion at T6 or above compared with patients with a lesion below T6 [33]. In patients with a lesion at T6 or above, a higher risk was observed in patients with higher lesion levels [33]. However, autonomic dysreflexia during the urodynamic investigation was observed also in patients with an NLI below T6. The risk of autonomic dysreflexia is two to three times higher in the presence of neurogenic detrusor overactivity at the urodynamic evaluation. Moreover, the observed prevalence of autonomic dysreflexia is three times higher in patients with complete SCI than in those with incomplete injury (91% versus 27%, respectively) [59]. These findings suggest the importance of integrating neurological and urodynamic data to identify patients at higher risk to develop autonomic dysreflexia.

## 4. Clinical and Research Implications

A reliable prognosis of genitourinary and gastroenteric outcomes is important for many aspects of clinical practice [60]. First, a reliable prognosis represents a fundamental prerequisite of patients’ and caregivers’ counseling about the expected functional outcomes. In addition, a reliable prognosis allows the team to establish realistic rehabilitative aims, which should be shared with patients and caregivers to increase motivation and compliance with the rehabilitative interventions. Finally, early prognosis enables timely planning of the interventions required for home and vocational reintegration of patients with SCI. Over the last years, various innovative treatments for neurogenic lower urinary tract dysfunction have been proposed and new promising perspectives are emerging from preclinical studies. To assess the efficacy of these new treatment options and to identify the category of patients who may most benefit from these interventions, clinical trials should be planned in the future [61]. On the other hand, the landscape of sexual and bowel rehabilitation is completely different. Advances in neurogenic sexual and bowel dysfunction treatment are poor, and the availability of limited knowledge about spontaneous evolution and validated rehabilitative protocols implies that the rehabilitative approaches to treat sexual and bowel dysfunction are mainly left to uncoordinated and empirical approaches [20,26,62].

The knowledge of spontaneous functional evolution and the availability of reliable prediction models is essential for the design of future randomized clinical trials, in order to balance the groups of interventions based on the expected recovery with standard therapy. Therefore, the availability of reliable models to predict lower urinary tract and bowel function may have important implications for research in these fields. On the other hand, the poor evidence regarding sexual function evolution and the lack of reliable prediction models should be considered a gap to be filled in future research.

## 5. Future Perspectives

To date, reliable prediction models of the lower urinary tract and bowel function are available, but future research to implement the actual models and increase the accessibility to these tools is desirable. For example, the addition of urodynamic parameters in models based only on clinical evaluation may enhance their predictive capacity. Moreover, the availability of simple online resources for the prediction may facilitate access to these tools for physicians of various specialties, general practitioners, professionals of the rehabilitative teams, patients, and caregivers, thus increasing awareness and access to a reliable prognosis.

Future refinement of available prediction models may be necessary in light of the change in the epidemiology of traumatic SCI, taking into consideration that the higher rate of comorbidities and medications of the elderly population may influence genitourinary and gastroenteric outcomes.

On the other hand, the lack of a reliable prediction model of sexual function represents an urgent gap to fill with the design of future research [62].

It is important to note that the available prediction models of lower urinary tract and bowel function were derived and validated in cohorts of patients who performed a rehabilitation program that included multidisciplinary treatment of lower urinary tract and bowel dysfunction and that patients were managed according to the European Association of Urology (EAU) Guideline on Neuro-Urology [63]. The validity of these models in patients who did not follow this management, for instance, patients from low-resourced countries who do not have access to urodynamic investigations or intermittent catheterization, remains to be proven [62].

The available knowledge on genitourinary and gastroenteric outcomes prediction was substantially derived from data of adult patients with traumatic SCI and validated in cohorts of patients with ischemic cause. These two etiologies are better studied and characterized from a functional point of view, due to the comparability in terms of single-event onset and clinical evolution [64]. However, the growing epidemiological relevance of non-traumatic etiologies imposes the need for validated outcome measures and prediction models also for patients with non-traumatic and non-ischemic SCI [11]. Finally, research efforts to generate reliable prediction models of genitourinary and gastroenteric outcomes should be implemented also for the pediatric population affected by SCI [65].

## 6. Conclusions

The application of available models allows a reliable prediction of lower urinary tract and bowel function after traumatic and ischemic SCI for clinical and research purposes. Further diffusion and refinement of these tools are desirable. There is an urgent need for prediction models of sexual function recovery to implement rehabilitation protocols and ameliorate future clinical trials in this field, which retains the utmost importance for patients’ health and quality of life. Future research should consider the need for reliable predictive models of genitourinary and gastroenteric outcomes also for patients with non-traumatic and non-ischemic SCI.

## Data Availability

Not applicable.
